# Concerted Regulation of cGMP and cAMP Phosphodiesterases in Early Cardiac Hypertrophy Induced by Angiotensin II

**DOI:** 10.1371/journal.pone.0014227

**Published:** 2010-12-03

**Authors:** Walid Mokni, Thérèse Keravis, Nelly Etienne-Selloum, Alison Walter, Modou O. Kane, Valérie B. Schini-Kerth, Claire Lugnier

**Affiliations:** CNRS UMR 7213, Biophotonique et Pharmacologie, Faculté de Pharmacie, Université de Strasbourg, Illkirch, France; University of Oldenburg, Germany

## Abstract

Left ventricular hypertrophy leads to heart failure and represents a high risk leading to premature death. Cyclic nucleotides (cAMP and cGMP) play a major role in heart contractility and cyclic nucleotide phosphodiesterases (PDEs) are involved in different stages of advanced cardiac diseases. We have investigated their contributions in the very initial stages of left ventricular hypertrophy development. Wistar male rats were treated over two weeks by chronic infusion of angiotensin II using osmotic mini-pumps. Left cardiac ventricles were used as total homogenates for analysis. PDE1 to PDE5 specific activities and protein and mRNA expressions were explored.

Rats developed arterial hypertension associated with a slight cardiac hypertrophy (+24%). cAMP-PDE4 activity was specifically increased while cGMP-PDE activities were broadly increased (+130% for PDE1; +76% for PDE2; +113% for PDE5) and associated with increased expressions for PDE1A, PDE1C and PDE5A. The cGMP-PDE1 activation by Ca^2+^/CaM was reduced. BNP expression was increased by 3.5-fold, while NOX2 expression was reduced by 66% and AMP kinase activation was increased by 64%. In early cardiac hypertrophy induced by angiotensin II, all specific PDE activities in left cardiac ventricles were increased, favoring an increase in cGMP hydrolysis by PDE1, PDE2 and PDE5. Increased cAMP hydrolysis was related to PDE4. We observed the establishment of two cardioprotective mechanisms and we suggest that these mechanisms could lead to increase intracellular cGMP: i) increased expression of BNP could increase “particulate” cGMP pool; ii) increased activation of AMPK, subsequent to increase in PDE4 activity and 5′AMP generation, could elevate “soluble” cGMP pool by enhancing NO bioavailability through NOX2 down-regulation. More studies are needed to support these assumptions. Nevertheless, our results suggest a potential link between PDE4 and AMPK/NOX2 and they point out that cGMP-PDEs, especially PDE1 and PDE2, may be interesting therapeutic targets in preventing cardiac hypertrophy.

## Introduction

Left ventricular hypertrophy is a major risk factor for premature death [1]. There is a correlation between plasmatic angiotensin II, hypertension, development of cardiac hypertrophy [2] and remodeling leading to heart failure [3]. Molecular mechanisms altered by angiotensin II have been mainly studied in vessels (for review see [4]); only a few studies have been performed in heart [5]. Cyclic nucleotides play a major role in the activation of different intracellular signaling pathways. In the heart, cyclic nucleotide phosphodiesterases (PDEs), by hydrolyzing cAMP and/or cGMP, regulate contractility in response to different stimuli such as β-adrenergic receptors [6] and natriuretic peptide receptors [7] and participate to cardiac remodeling [8].

PDE superfamily is constituted of eleven families, PDE1 to PDE11, but only the five former have been well studied [9]. PDE1 is activated by calmodulin (CaM) in presence of Ca^2+^ (PDE1-Ca^2+^/CaM complex). PDE1A and PDE1B hydrolyze mainly cGMP, while PDE1C hydrolyzes equally cAMP and cGMP. The activations of PDE1A and PDE1C by Ca/CaM are decreased by PKA phosphorylation. PDE2 hydrolyzes cGMP and cAMP, and its cAMP hydrolysis is allosterically activated by cGMP [10]. PDE3 hydrolyzes both cAMP and cGMP. cGMP has a greater affinity for PDE3 while being hydrolyzed 10 times slower than cAMP, which makes it acts as a PDE3 inhibitor. PDE4 hydrolyzes specifically cAMP and could be activated subsequently through PKA-dependent phosphorylation while PDE5 hydrolyzes specifically cGMP and could be activated by cGMP and PKG-dependent phosphorylation [11].

Some studies have shown changes in the activity, expression and distribution of PDEs in various forms of cardiac hypertrophy [12], [13] and heart failure [14], [15]. Indeed, a decrease in cAMP-PDE activity, especially PDE3A activity, was observed in a^2+^dvanced cardiac hypertrophy induced by ligation of the aorta in mice as well as in human failing hearts [16]. In dog, heart failure was associated with a decrease in membrane expression of PDE3, without change in cytosolic PDE3 activity [17]. Furthermore, inactivation of the PDE4D gene in mouse was associated with progressive heart failure and arrhythmias, despite a normal overall cAMP level [14]. These results are consistent with the observation that, in spontaneously hypertensive rats subjected to a chronic β-adrenergic stimulation *in vivo,* pentoxyfilline (a non-selective PDE inhibitor) promotes the transition from left ventricular hypertrophy to left ventricular dilatation [18]. In cardiomyocytes, cGMP has opposite effects depending on its compartmentation. The elevation of cGMP due to the stimulation of particulate guanylyl cyclase (GC) by natriuretic peptides cause positive inotropic and chronotropic effects [19], mediated by cGMP/cAMP cross-talk [10]. In contrast, a permeable cGMP analogue [6] or the stimulation of GC by NO donors through activation of PKG causes a negative inotropic effect via the myofilaments [20]. It was interestingly shown that PDE5A was upregulated in different models of cardiac disease [15], [21] and that early correction of PDE5 alterations could restore heart function and prevent cardiac hypertrophy [22], [23].

Therefore, PDEs in the heart could be valuable therapeutic targets for the treatment and prevention of cardiac dysfunction. Since no studies were done investigating simultaneously the various cAMP- and cGMP-PDE isoform contributions in cardiac pathology, we aimed to explore the variations of PDE1 to PDE5 in left cardiac ventricle on a rat model of cardiac hypertrophy induced by angiotensin II, and especially in the initial stages of cardiac hypertrophy development.

## Materials and Methods

### In vivo Treatment of Rats

The study conforms to the *Guide for the Care and Use of Laboratory Animals* published by the US National Institute of Health (NIH Publication No. 85-23, revised 1996) and has been approved by the local ethics committee of animal experimentation (CREMEAS). Experimental protocol and physiological measurements were conducted as previously described [24]. Male Wistar rats (10-week old) were anesthetized with sodium pentobarbital (50 mg/kg, i.p.; Centravet). A 1-cm incision was made in the mid-scapular region and an osmotic mini-pump (Alzet 2004, Charles River Laboratories) was implanted. Pumps contained angiotensin II (Sigma-Aldrich, St. Louis, MO, USA), which was dissolved in saline solution, and the infusion rate was 0.1 mg.kg^−1^.d^−1^ (ANGII 0.1) or 0.4 mg.kg^−1^.d^−1^ (ANGII 0.4) for 14 days. Sham-operated rats underwent an identical surgical procedure without pump implantation. Systolic blood pressure and heart rate were measured using a tail-cuff plethysmograph connected to a computerized system (LE 5002, BIOSEB) in conscious rats before the infusion (D0) and at the end of treatment (D14). Body weight was also recorded at D0 and D14. Rats were then sacrificed, hearts were weighted and left cardiac ventricles (LCV) were quickly frozen in liquid nitrogen and stored at −80°C.

### Preparation of Protein Extracts from Left Cardiac Ventricles

Frozen left ventricles were powdered in liquid nitrogen with a freezermill (Freezer/Mill 6750 from SPEX CERTIPREP INC). Grinding protocol included 5 cycles (1 min freezing, 2 min grinding with 1 min freezing between two grindings). Powders were suspended (100 mg of tissue/ml) in a protease inhibitor buffer (20 mM Tris, 2 mM Mg^2 +^ acetate, 5 mM EGTA, pH 7.5, 1 mM dithiothreitol, 10 µg/ml aprotinin, 10 µg/ml leupeptin, 10 µg/ml soybean trypsin inhibitor, 0.33 mM Pefabloc). Homogenates were then mixed and treated by ultrasounds for 10 seconds. Total homogenates obtained were aliquoted and stored at −80°C until use. Protein levels were assessed by the Lowry method [25] using bovine serum albumin (BSA) as standard. DNA levels were determined by fluorometric measurement as previously described [26] using a DNA standard provided by Sigma.

### Western Blot Analysis

Proteins (30 µg) from left cardiac ventricle homogenates were subjected to Western blot as described previously [27]. Immunodetection was carried out with: anti-PDE1A (PD1A-101AP; 1/2,500), anti-PDE1B (PD1B-201AP; 1/2,500), anti-PDE1C (PD1C-301AP; 1/2,500), anti-PDE2A (PD2A-101AP; 1/1,000), anti-PDE4C (PD4-301AP; 1/2,500), anti-PDE4D (PD4-401AP; 1/2,500), anti-PDE5A (PD5A-101AP; 1/2,500) and anti-phospho-specific PDE5A (PPD5-140AP; 1/1,000; labels PDE5A proteins when phosphorylated at Ser92 by protein kinase G (PKG)) from FabGennix Inc. (Frisco, TX, USA); anti-PDE4A (AC55;1/2,000) and anti-PDE4B (K118; 1/2,000) from Dr M. Conti [28]; anti-phospho-AMPKα (Thr172) subunit (2531; 1/1,000) from Cell Signaling Technology, Inc.; anti-p47-phox (sc-14015; 1/,1000) from Santa Cruz Biotechnology, Inc.; anti-NOS3 (610297; 1/2,000) from BD Transduction Laboratories; anti-GAPDH (1/60,000; Chemicon). Immobilized antigens were detected by chemiluminescence using horse radish peroxidase-conjugates as secondary antibodies (1/60,000; Promega), an ECL kit (GE Healthcare) and autoradiography films. Autoradiography signals were captured on a GeneGenius Bio Imaging System (Syngene) using the GeneSnap software and analysed using the GeneTool software. PDE signal values were normalized with GAPDH signal values.

### PDE Assay

PDE activity was determined with a radioenzymatic assay as described previously [29]. Total cAMP-PDE activity was assessed at 1 µM cAMP and the contribution of PDE isozymes was determined by using selective inhibitors: 1 µM cilostamide for PDE3 (K_i_ = 45 nM [30]) and 10 µM rolipram for PDE4 (K_i_ = 0.8 µM [30]), the residual cAMP-PDE activity representing essentially PDE2 [29]. Total cGMP-PDE activity was assessed at 1 µM cGMP and the contribution of PDE isozymes was determined by using the selective inhibitors: 10 µM nimodipine for PDE1 (Ki = 1.4 µM [30]), 20 µM EHNA for PDE2 (IC_50_ = 0.8–2 µM [31]) and 0.1 µM DMPPO for PDE5 (Ki = 3 nM [32]). The residual cGMP-PDE activity represented essentially PDE3, which is able to hydrolyze both cAMP and cGMP, but with a higher affinity and a 10-fold lower V_max_ for cGMP [29]. As EHNA is effective only on cGMP-stimulated PDE2 and not on basal PDE2 [33], it was not usable to determine cAMP-PDE2 activity. Specific activities were expressed as pmol.min^−1^.mg^−1^ protein and for some experiments also as pmol.min^−1^.mg^−1^ DNA.

### Quantitative real-time RT-PCR

Total RNA was isolated from left cardiac ventricle using the RNeasy® kit (Qiagen), including an RNase-free DNase treatment to prevent co-amplification of genomic DNA. The concentration of the purified RNA was determined spectrophotometrically at 260 nm. The integrity of purified RNA was checked by agarose gel electrophoresis in denaturing condition. The reverse transcription was performed using iScript® cDNA synthesis kit from Bio-Rad. The 18S rRNA house-keeping gene was used for normalization. Specific primers for PDEs and 18S rRNA were designed with Primer3 (v. 0.4.0) software using mRNA sequences retrieved from NCBI nucleotide database and are presented in Table 1. The transcript levels of PDEs and 18S rRNA were quantified by real-time PCR performed in the MyiQ® single-color detector system (Bio-Rad Laboratories, Inc.) using the iQ SYBR® Green Supermix (product no. 170-8884) containing the hot-start iTaq DNA polymerase. The amplification conditions were: 95°C for 5 min, 40 cycles at 94°C for 10 sec, 60°C for 10 sec and 72°C for 15 sec. All amplification efficiencies were 1.0±0.1. Specificity of amplification products was assessed by melting curve analysis and their size was checked by agarose gel electrophoresis. Samples were processed in triplicate according to the manufacturer's guidelines. Relative gene expression was calculated using the comparative threshold (Ct) method (2^−ΔΔ*C*t^) [34].

**Table 1:** Primer sequences used for Real-Time PCR experiments.

		Sequence	Product size (pb)	GenBank accession no.
**PDE1A**	Forward	5′-ACCATGATTGGGTTCCATGTT-3′	175	NM_030871
	Reverse	5′-CAGCCAACTCTTTCCACCT-3′		
**PDE1B**	Forward	5′-TCCACATCCAGACCAAGTCA-3′	194	NM_022710
	Reverse	5′-GCAGGACATGTCTGTGGCT-3′		
**PDE1C**	Forward	5′-CAGCCTACCGTCTTCTCCA-3′	179	NM_031078
	Reverse	5′-TTCAATTGCTTCTGGTTGCTG-3′		
**PDE2A**	Forward	5′-AGTGCTGGGAGAAGAGGTC-3′	181	NM_031079
	Reverse	5′-GTCAGTGGCTCGACTGATG-3′		
**PDE3A**	Forward	5′-GCCCCAGTGTTGATGACTCT-3′	161	NM_017337
	Reverse	5′-GGTGATCCTTGAGGAGGTGA-3′		
**PDE3B**	Forward	5′-ATGGAATTCAAGCGTTTTCG-3′	169	NM_017229
	Reverse	5′-TGCACACCTGGCAGACTAAG-3′		
**PDE4A**	Forward	5′-GAAGACAACCGGGACTGGT-3′	172	NM_013101
	Reverse	5′-CCTCAGTGGTAGGCAATCC-3′		
**PDE4B**	Forward	5′-CCTCCGACACCTTCGTAAC-3′	153	NM_017031
	Reverse	5′-CCAGGTCTGTGAAGACAGC-3′		
**PDE4C**	Forward	5′-GAAGGGCACTACCACTCCA-3′	153	XM_001070301
	Reverse	5′-GTGTATAGCGCACGCAAAGA-3′		
**PDE4D**	Forward	5′-TGGGCAGACCTCGTACATC-3′	190	NM_017032
	Reverse	5′-CAGTGTCTGACTCGCCATC-3′		
**PDE5A**	Forward	5′-GGGAAGAGGTCGTTGGTGT-3′	162	NM_133584
	Reverse	5′-TTTGTTCTCCAGCAGTGACG-3′		
**NOX1**	Forward	5′-CATGGATGGATCTCTTGCCT-3′	168	NM_053683
	Reverse	5′-ACCATGAGAACCAAAGCCAC-3′		
**NOX2**	Forward	5′-AACGTGGAGTGGTGTGTGAA-3′	222	AF298656
	Reverse	5′-TTTGGTGGAGGATGTGATGA-3′		
**NOX3**	Forward	5′-AGTGAACAAGGGAAGGCTCA-3′	176	AY573239
	Reverse	5′-GCAATCTGCTTGAATTCCTCA-3′		
**NOX4**	Forward	5′-TGTCTGCATGGTGGTGGTAT-3′	154	NM_053524
	Reverse	5′-ATACTTCAACAAGCCACCCG-3′		
**p47phox**	Forward	5′-AGCTCCCAGGTGGTATGATG-3′	205	AY029167
	Reverse	5′-ATCTTTGGCCGTCAGGTATG-3′		
**NOS1**	Forward	5′-CTGCAAAGCCCTAAGTCCAG-3′	207	NM_052799
	Reverse	5′-AGTGTTCCTCTCCTCCAGCA-3′		
**NOS2**	Forward	5′-AGGGAGTGTTGTTCCAGGTG-3′	232	NM_012611
	Reverse	5′-TCCTCAACCTGCTCCTCACT-3′		
**NOS3**	Forward	5′-TGTGACCCTCACCGATACAA-3′	212	NM_021838
	Reverse	5′-CTGGCCTTCTGCTCATTTTC-3′		
**ANP**	Forward	5′-AGCGAGCAGACCGATGAA-3′	161	M27498
	Reverse	5′-GCCCTCAGTTTGCTTTTCAA-3′		
**BNP**	Forward	5′-CAGCTCTCAAAGGACCAAGG-3′	191	M25297
	Reverse	5′-TAAAACAACCTCAGCCCGTC-3′		
**CNP**	Forward	5′-GGCAATCAGAAAAAGGGTGA-3′	185	D90219
	Reverse	5′-CCTTTGGACAAGCCCTTCTT-3′		
**18S rRNA**	Forward	5′-AAACGGCTACCACATCCAAG-3′	155	M11188
	Reverse	5′-CCTCCAATGGATCCTCGTTA-3′		

### Immunohistochemistry

Frozen left cardiac ventricles embedded in Tissue-Tek® O.C.T (Sakura Finetek) were cryosectioned (4 µm) and fixed with 4% paraformaldehyde. Fixed sections were incubated with antibodies directed against PDE5A (1/100; FabGennix Inc.). The corresponding IgG coupled to Alexa 488 (Invitrogen, Molecular Probes) was used as secondary antibody. Images were obtained with a Leica DM 400 inverted epifluorescence microscope (x63 objective).

### Statistical Analysis

All data are expressed as mean ± S.E.M. Statistical analyses were performed with Student's *t* test for unpaired data excepted for PDE4 enzymatic activities measured with H89 (paired data), using GraphPad Prism software (San Diego, USA). For Real-Time PCR, statistical analyses were performed with Student's *t* test for unpaired data as described previously [35]. A value of *P*<0.05 was considered as significant.

## Results

### Effect of angiotensin II treatment on cardiovascular parameters

**Figure 1:**
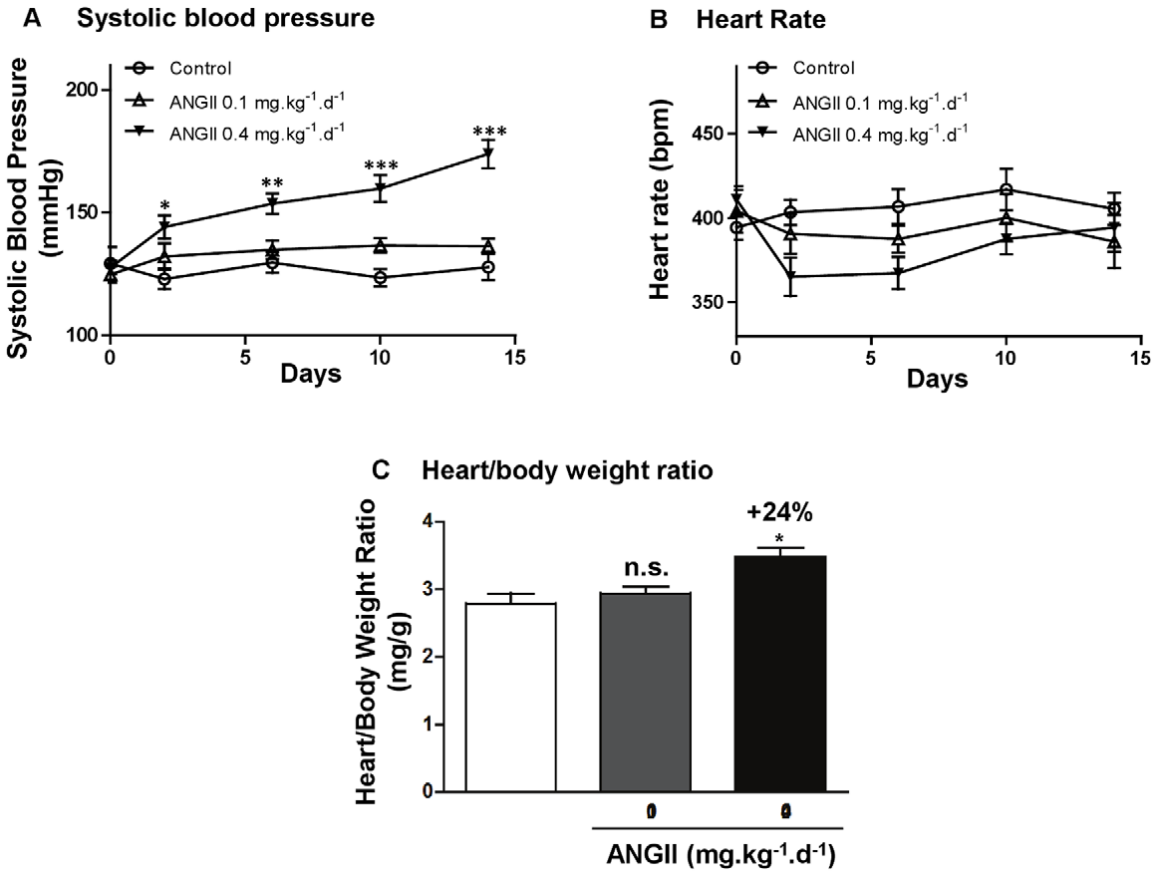
Effects of angiotensin II treatment on rat physiological parameters. Systolic blood pressure (**A**), heart rate (**B**) and heart/body weight ratio (**C**). Rats were treated with angiotensin II at 0.1 mg.kg^−1^.d^−1^ (ANGII 0.1; n = 8) or 0.4 mg.kg^−1^.d^−1^ (ANGII 0.4; n = 13). Effects were expressed in comparison with control rats (n = 6). n.s.: not significant; *: *P*<0.05; **: *P*<0.01; ***: *P*<0.001.

Before the implantation of mini-pumps, rats presented similar systolic blood pressures (129±7 mmHg for control rats, 125±3 mmHg for 0.1 mg.kg^−1^.d^−1^ and 128±3 mmHg for 0.4 mg.kg^−1^.d^−1^ angiotensin II treated rats). After two week-treatment, systolic blood pressure was significantly increased (+36%, *P*<0.001) only in rats treated with 0.4 mg ANGII (174±5 mm Hg) compared to control rats (128±5 mm Hg) (**Fig. 1A**). Heart rate did not change significantly among the three groups (**Fig. 1B**). The heart/body weight ratio was calculated as an indicator for estimating cardiac hypertrophy. The development of a slight cardiac hypertrophy (+24%, *P* = 0.02, **Fig. 1C**) was observed only in rats treated with 0.4 mg ANGII. This dose was kept for the forthcoming analysis.

### PDE isozyme distribution in left cardiac ventricle

**Figure 2:**
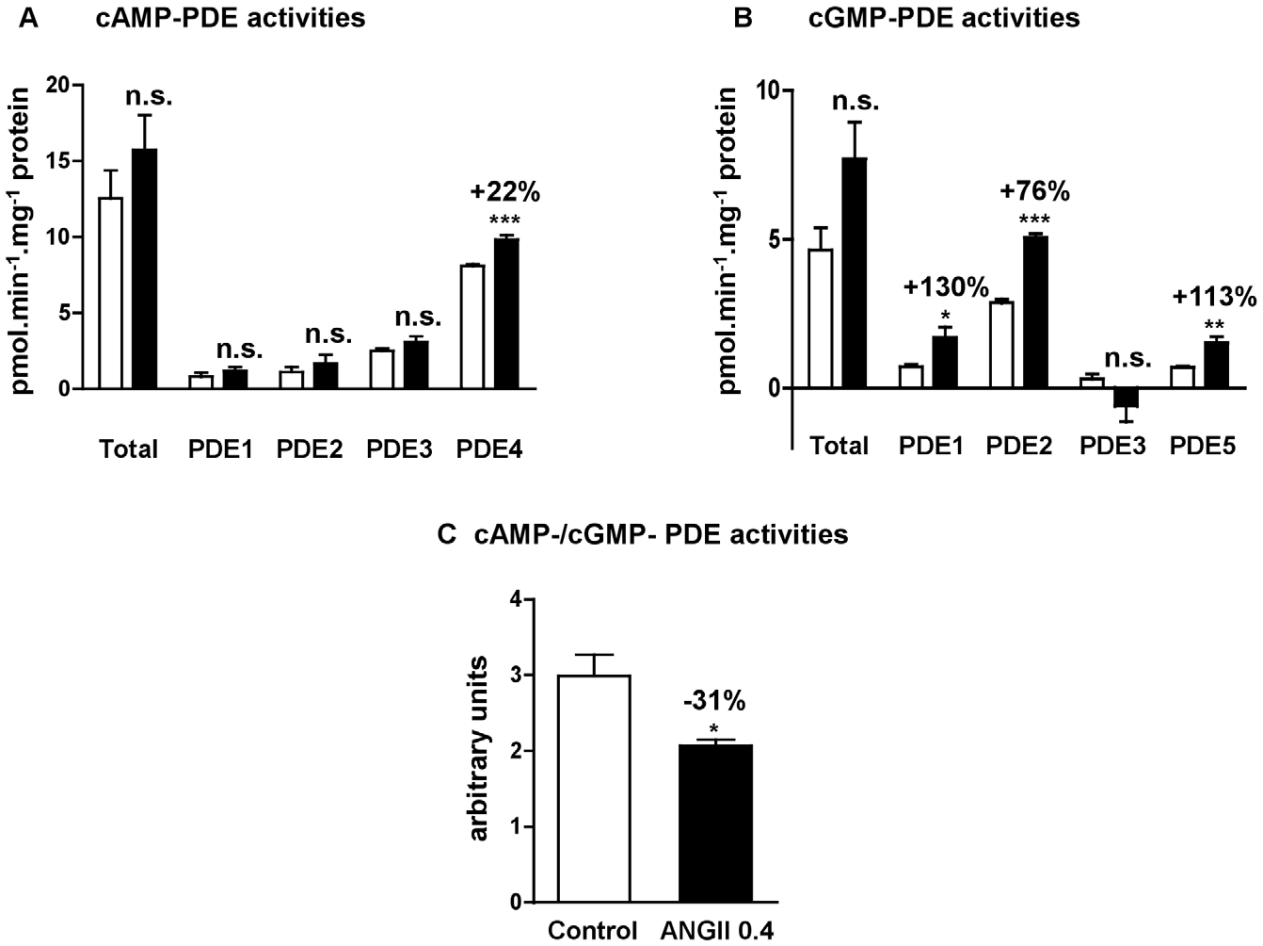
Effects of angiotensin II treatment on left cardiac ventricle PDE specific activities. cAMP-PDE specific activities and contribution of PDE2, PDE3 and PDE4 (**A**). cGMP-PDE specific activities and contributions of PDE1, PDE2, PDE3 and PDE5 (**B**). cAMP-PDE over cGMP-PDE total activity ratio (**C**). Specific activities were determined on total homogenate and expressed as pmol.min^−1^.mg^−1^ of protein. Effects on treated rats (▪; n = 4) were compared with control rats (□; n = 6). n.s.: not significant; *: *P*<0.05; **: *P*<0.01; ***: *P*<0.001.

**Figure 3:**
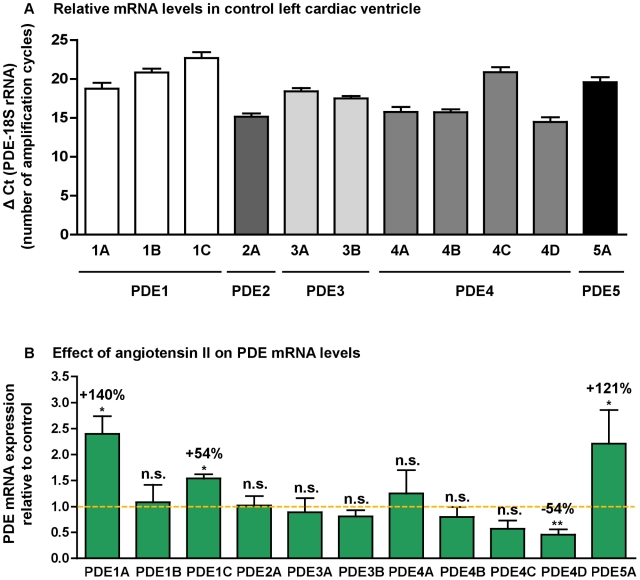
PDE mRNA distribution in control left cardiac ventricle and effect of angiotensin II treatment. Relative mRNA levels in control left cardiac ventricle (**A**). Results were expressed as ΔCt  =  Ct of PDE – Ct of 18S rRNA, which corresponds to the number of amplification cycles needed to detect fluorescence signal, each cycle corresponding to a 2-fold amplification. ΔCt being inversely proportional to initial PDE mRNA level, the fewer is ΔCt, the greater is initial mRNA level. Effects of angiotensin II treatment on PDE mRNA expressions (**B**). Results were normalized with the 18S rRNA housekeeping gene and expressed as amplification folds relative to control. n.s.: not significant; *: *P*<0.05; **: *P*<0.01.

In left cardiac ventricle of control rats, PDE4 represented 64% of total cAMP-PDE activity whereas PDE1, PDE2 and PDE3 contributed respectively for 7%, 9% and 20% (**Fig. 2A**). PDE2 represented 62% of total cGMP-PDE activity, whereas PDE1, PDE3 and PDE5 contributed, respectively for 15%, 7% and 16% for total cGMP-PDE activity (**Fig. 2B**). This pattern is in accordance with the PDE mRNA distribution in control left cardiac ventricle, where PDE2A, PDE4A, PDE4B and PDE4D are the major PDE transcripts, followed by PDE3A, PDE3B, PDE5A and PDE1A at almost the same level, and finally PDE4C and PDE1C are the least expressed (**Fig. 3A**).

### Effect of angiotensin II treatment on PDE activities

As seen in **Figure 1**, rats treated with angiotensin II at 0.4 mg.kg^−1^.d^−1^ for two weeks showed arterial hypertension associated with cardiac hypertrophy, without developing tachycardia**.** Although this treatment did not significantly change total cAMP-PDE activity, it induced a specific 22%-increase in PDE4 activity (*P*<0.001) (**Fig. 2A**). On the other side, cGMP-PDE activities were broadly increased, for PDE1 (+130%, *P* = 0.02), PDE2 (+76%, *P*<0.001) and PDE5 (+113%, P = 0.002) (**Fig. 2B**). Moreover, the total cAMP-PDE/cGMP-PDE activity ratio was decreased by 31% in hypertrophic left cardiac ventricles, indicating a shift to a greater cGMP hydrolysis (**Fig. 2C**).

Because cardiac hypertrophy induces alterations in cellular protein content and could thus introduce a bias in the measurement of PDE activity expressed as pmol.min^−1^.mg^−1^ of protein, PDE activity was also expressed as pmol.min^−1^.mg^−1^ of DNA. We observed similar effects: cAMP-PDE4 activity was increased (+13%), as were cGMP-PDE1 (+105%), cGMP-PDE2 (+60%) and cGMP-PDE5 (+61%) (not illustrated).

### Exploration of increased cAMP hydrolysis activity

**Figure 4:**
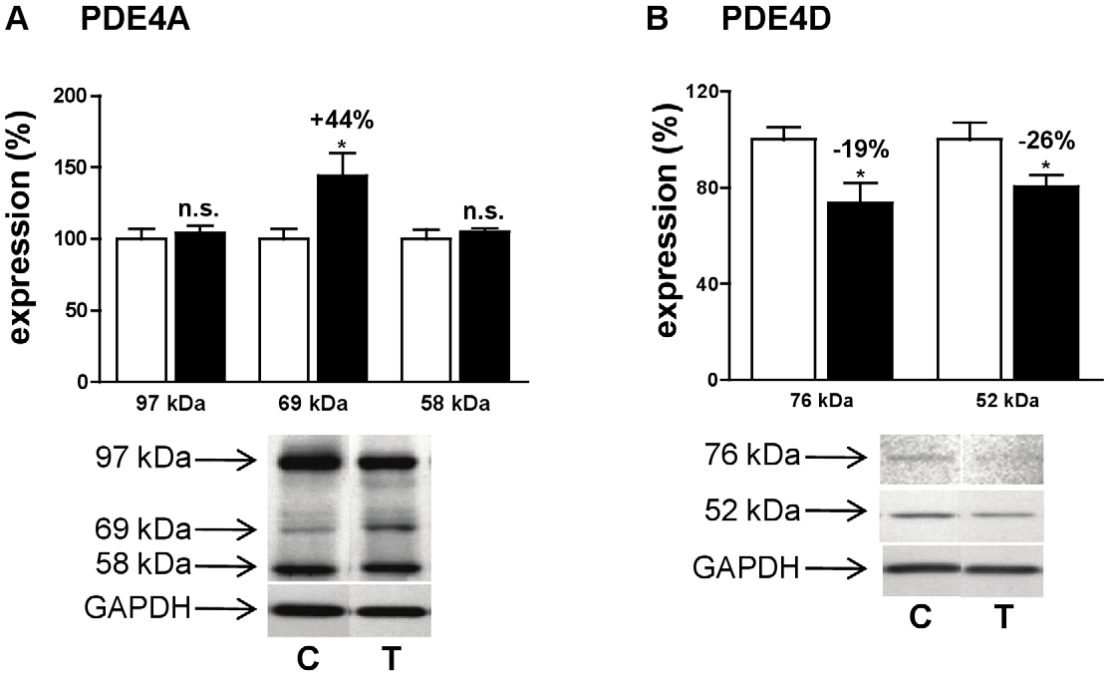
Effect of angiotensin II treatment on cAMP hydrolysis activity. Effects of angiotensin II treatment at 0.4 mg.kg^−1^.d^−1^, on protein expression of PDE4A (**A**), PDE4D (**B**). Effects on treated rats (T, ▪; n = 4) were compared with control rats (C, □; n = 4). Results were normalized with GAPDH signal and expressed in percentage of untreated rat. n.s.: not significant; *: *P*<0.05.

Considering the increased PDE4 activity, the expression of PDE4 subtypes has been explored. Although PDE4A mRNA was not significantly modified (**Fig. 3B**), Western blot revealed a specific 44%-increase in a 69 kDa-PDE4A variant (**Fig. 4A**) among the three variants expressed (97 kDa, 69 kDa and 58 kDa). No significant changes for PDE4B and PDE4C mRNA (**Fig. 3B**) and protein (not illustrated) expressions were observed. Oppositely, angiotensin II treatment induced significant decreases in PDE4D variant expressions (−19% for 76 kDa and −26% for 52 kDa, **Fig. 4B**) and PDE4D transcript (−54%, **Fig. 3B**). PDE3A and PDE3B mRNA expressions were not significantly changed (**Fig. 3**).

### Effect of angiotensin II treatment on calmodulin-dependent activation of PDE1

**Figure 5:**
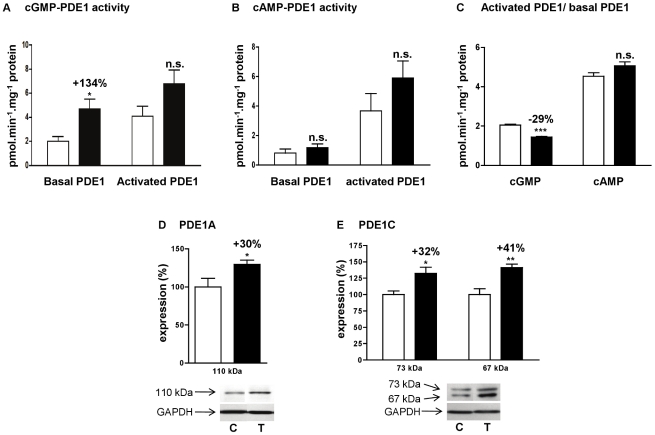
Effect of angiotensin II treatment on PDE1 activity in left cardiac ventricle. cGMP-PDE1 specific activity (**A**), cAMP-PDE1 specific activity (**B**), and PDE1 activated over basal activity ratio (**C**). Basal PDE1 activity was assessed in presence of EGTA and CaM-activated PDE1 was assessed in presence of Ca^2+^ and CaM. Effects on treated rats (T, ▪; n = 4) were compared with control rats (C, □□; n = 6). Specific activities were determined on total homogenate and expressed as pmol.min^−1^.mg^−1^ of protein. Effects of angiotensin II treatment on protein expression of PDE1A (**D**) and PDE1C (**E**). Results were normalized with GAPDH signal and expressed in percentage of untreated rat. n.s.: not significant; *: *P*<0.05; **: *P*<0.01; ***: *P*<0.001.

The main characteristic of PDE1 is to be activated by intracellular calcium concentration increases through association with Ca^2+^/calmodulin (CaM) complexes. To explore the increase in cGMP-PDE1 activity reported in **Figure 2B**, we compared basal PDE1 activity (assessed in presence of EGTA) and CaM-activated PDE1 (assessed in presence of Ca^2+^ and CaM). As seen above, basal cGMP-PDE1 activity was significantly increased by 134% in ANGII 0.4 rats. But this increase was no longer significant when PDE1 was activated, by Ca^2+^/CaM complexes, indicating a loss in CaM-dependent activation (**Fig. 5A**). The ratio between activated and basal cGMP-PDE1 activities was significantly decreased by 29% (**Fig. 5C**), suggesting that PDE1 activation capacity was more important in control rats that in ANGII 0.4 rats. PDE1C hydrolyzing both cAMP and cGMP, the same approach was done for cAMP-PDE1 activity (**Fig. 5B**). No significant change in the CaM-dependent activation was observed between control and ANGII 0.4 rats (**Fig. 5C**), suggesting that different PDE1 families contribute to PDE1 activity.

Besides, angiotensin II treatment induced strong increases of PDE1 expressions. PDE1A mRNA was increased by 140% (**Fig. 3B**), associated with a 30% increase of 110 kDa-PDE1A protein expression (**Fig. 5D**). PDE1C mRNA, that was increased by 54% (**Fig. 3B**), was associated with an increased expression of the two detected variants (+41% for the 67 kDa signal and +32% for the 73 kDa signal) (**Fig. 5E**). PDE1B mRNA was detected by real-time PCR without being affected by angiotensin treatment (**Fig. 3B**) but PDE1B protein was not detected by Western blot.

### Effect of angiotensin II treatment on PDE5

**Figure 6:**
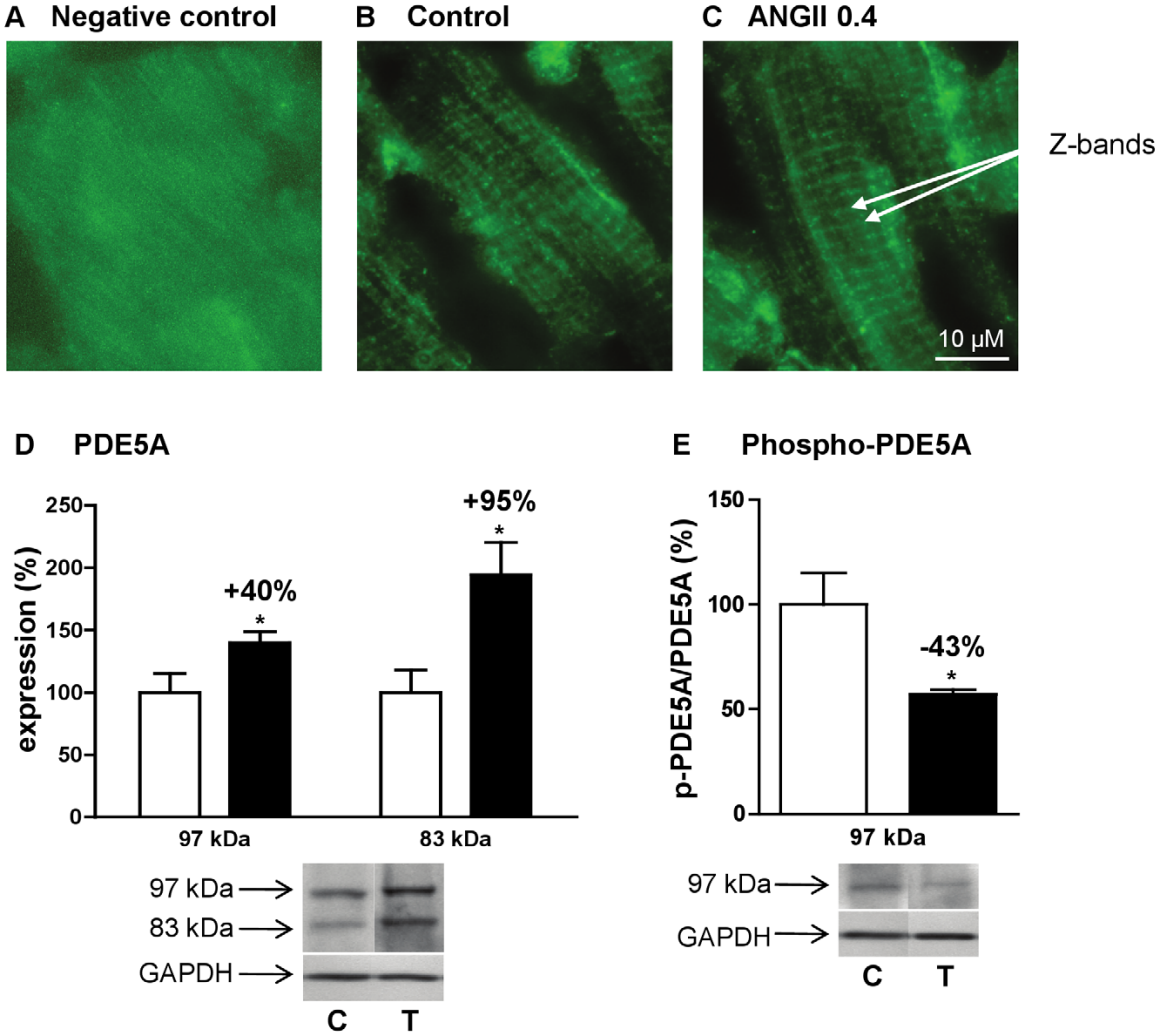
Effects of angiotensin II treatment on PDE5 expression and intracellular localization. Immunostaining of PDE5A cellular distribution in left cardiac ventricle: negative control (**A**), control rats (**B**) and angiotensin II treated rats (**C**). PDE5A is localized to Z-bands. Effects of angiotensin II treatment at 0.4 mg.kg^−1^.d^−1^ on protein expression of PDE5A (**D**) and phospho-PDE5A over 97 kDa PDE5A (**E**). Effects on treated rats (T, ▪; n = 4) were compared with control rats (C, □; n = 4). Results were normalized with GAPDH signal and expressed in percentage of untreated rat. *: *P*<0.05.

PDE5 was subjected to the most important changes in expression after angiotensin II treatment. PDE5A mRNA increased by 121% (**Fig. 3B**) and was associated with an increase of the two detected PDE5 variants: + 95% for 97 kDa-signal and +40% for 83 kDa-signal (**Fig. 6D**). Furthermore, Ser92-phophosphorylated 97 kDa-PDE5A/PDE5A 97 kDa-protein ratio was reduced by 43%, indicating a decrease in PDE5A activation by PKG (**Fig. 6E**). We also looked at the intracellular localization of PDE5A. Previous work reported that PDE5A was localized to Z-bands and that this localization was lost in advanced cardiac hypertrophy [36]. In our model of early cardiac hypertrophy, PDE5A localization to Z-bands (**Fig. 6B**) was not affected by angiotensin II treatment (**Fig. 6C**).

### PDE2 and natriuretic peptides

**Figure 7:**
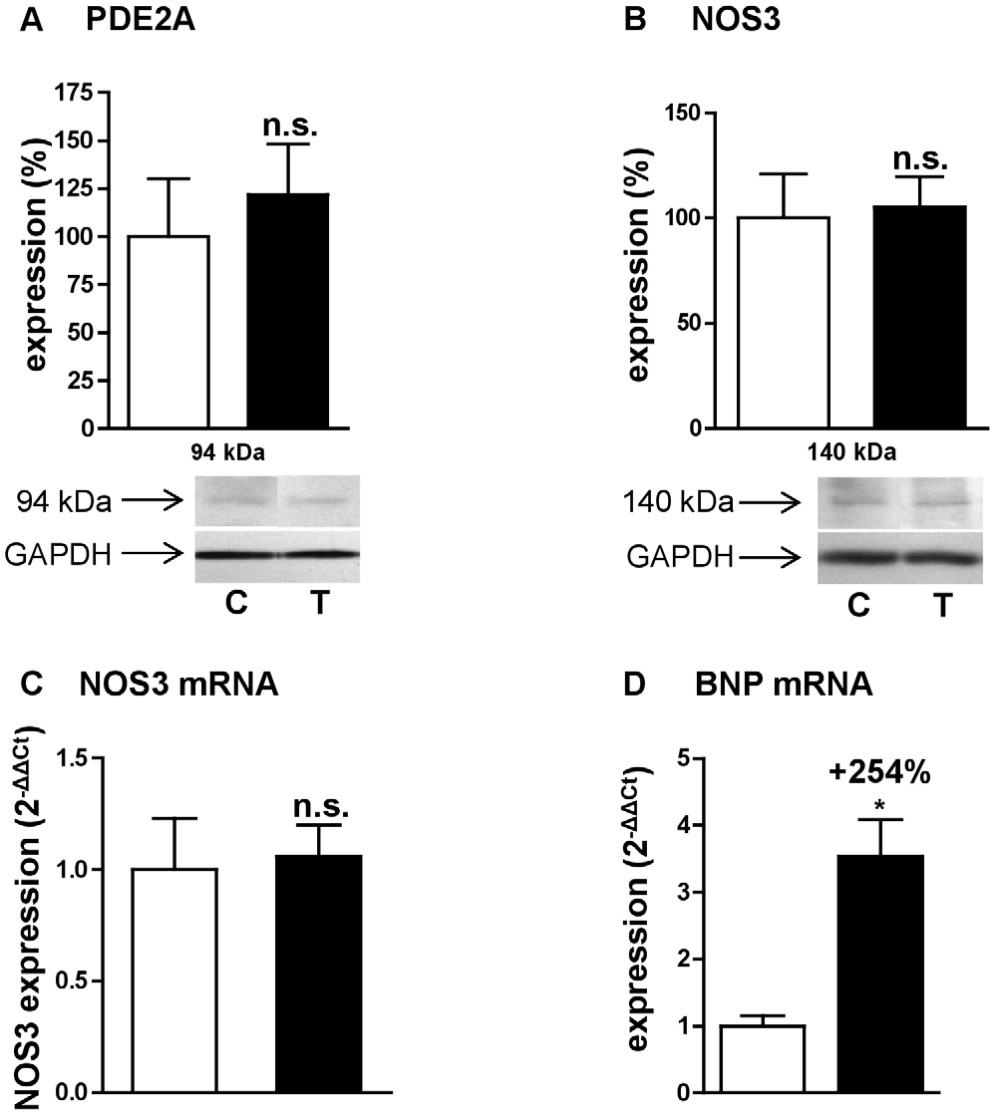
Effects of angiotensin II treatment on PDE2, BNP and NOS. Expression of PDE2A protein (**A**), NOS3 protein (**B**), NOS3 mRNA (**C**) and BNP mRNA (**D**). Effects on treated rats (T, ▪; n = 4) were compared with control rats (C, □; n = 4). Western blot results were normalized with GAPDH signal and expressed in percentage of untreated rat. Q-RT-PCR results were normalized with the 18S rRNA housekeeping gene and expressed as amplification folds relative to control. n.s.: not significant; *: *P*<0.05.

cGMP is produced in part by natriuretic peptide receptors. Natriuretic peptides (ANP, BNP and CNP) are synthesized in the heart and can exert their effect through an autocrine/paracrine pathway. Moreover, natriuretic peptide production in heart is increased in cardiac hypertrophy [7]. We therefore studied the expression of the natriuretic peptide transcripts in early cardiac hypertrophy induced by angiotensin II. While ANP and CNP transcripts were not significantly modified (not illustrated), BNP transcript was significantly increased by 254% (**Fig. 7D**).

On the other hand, despite an important increase in cGMP-PDE2 hydrolysis activity (**Fig. 2B**), angiotensin II treatment did not induce any change either in PDE2A mRNA expression (**Fig. 3B**) or in PDE2A protein expression (**Fig. 7A**).

### Effect on AMPK

**Figure 8:**
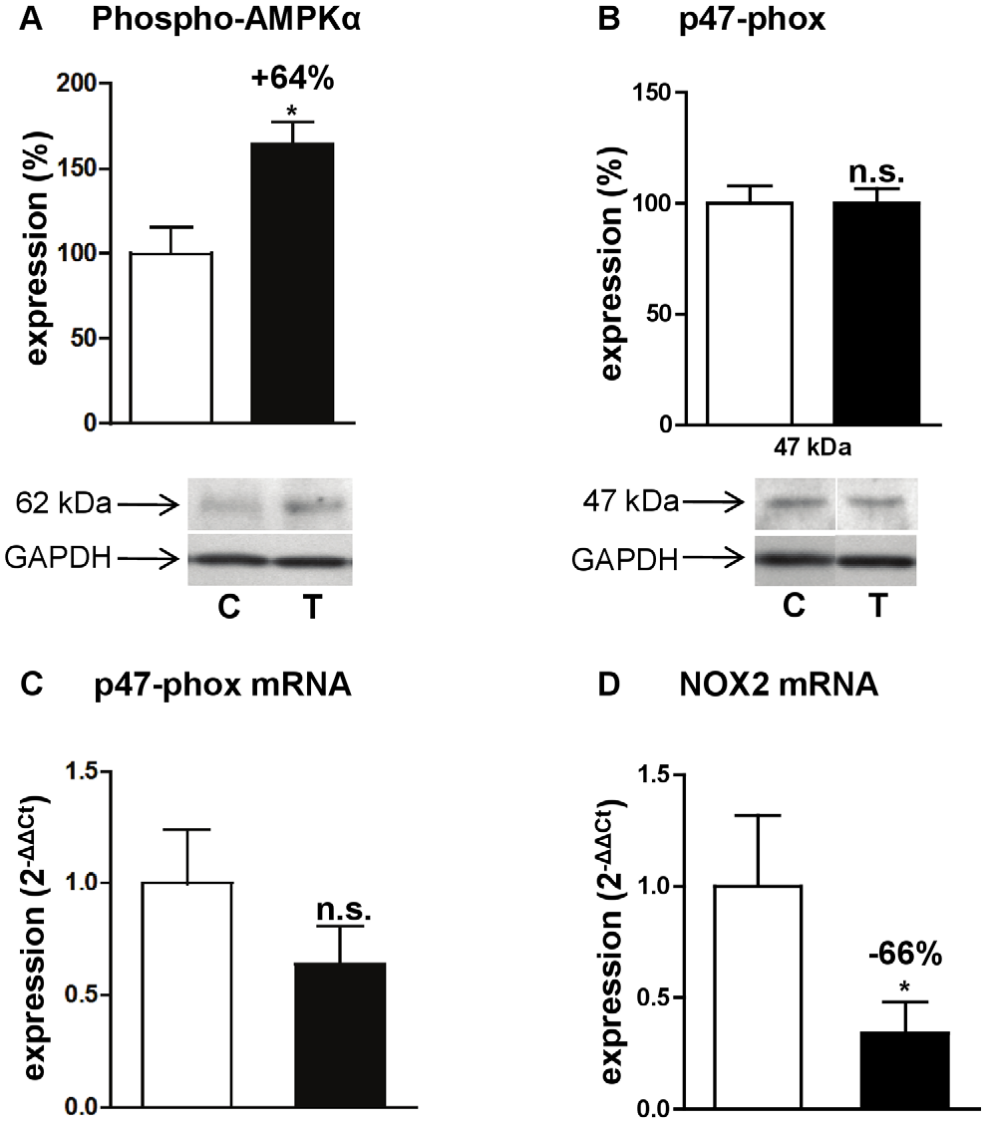
Effects of angiotensin II treatment on AMPK and NADPH oxidase. Expressions of the phosphorylated α-catalytic subunit of AMPK (**A**), p47-phox protein (**B**), p47-phox mRNA (**C**) and NOX2 mRNA (**D**). Effects on treated rats (T, ▪; n = 4) were compared with control rats (C, □; n = 4). Western blot results are expressed in percentage after correcting PDE signal with GAPDH signal. Q-RT-PCR results were normalized with the 18S rRNA housekeeping gene and expressed as amplification folds relative to control. n.s.: not significant; *: *P*<0.05.

5′AMP kinase (AMPK) plays a very important role in heart energy metabolism and protein synthesis. It produces ATP from AMP and is activated when the AMP/ATP ratio increases, i.e. under conditions of stress [37]. As we observed an increased cAMP hydrolysis into 5′AMP, we looked for AMPK activation. Consistently, an increase of 64% of the Thr172-phosphorylated AMPKα catalytic subunit was observed (**Fig. 8A**).

### Effect on nitric oxide synthase (NOS) expression

Another source of cGMP is that produced by soluble guanylyl cyclases in response to NO. Therefore, NOS1, NOS2 and NOS3 expressions were examined in left cardiac ventricle. Early cardiac hypertrophy induced by angiotensin II did not change either NOS1 or NOS2 mRNA expressions (not illustrated), NOS3 mRNA expression (**Fig. 7C**) or NOS3 protein expression (**Fig. 7B**).

### Effect on NADPH oxidases

Reactive oxygen species (ROS) have been shown to be implicated in the development of cardiac hypertrophy [38]. Angiotensin II modulates ROS production through NADPH oxidase (NOX) activity and expression in heart [39]. We studied therefore the effect of angiotensin II-induced early cardiac hypertrophy on the three major NOX enzymes present in the heart, NOX1, NOX2 (or gp91-phox) and NOX4 [40]. We found a significant 66% decrease in the mRNA expression of NOX2 (**Fig. 8D**), while NOX1 and NOX4 mRNA expressions were unchanged (not illustrated). Activation of NOX2 is known to need different subunits, and notably p47-phox subunit [38], but we found no change either in mRNA expression (**Fig. 8C**) or in protein expression (**Fig. 8B**) of p47-phox.

## Discussion

cAMP- and cGMP-PDE activities, previously determined in our laboratory on isolated cardiac myocytes from left cardiac ventricle of adult rats, showed that cAMP hydrolysis activity was almost 3 times greater than that of cGMP and that PDE2 and PDE4 were the major enzymes implicated [41]. The present results in left cardiac ventricles of control rats are consistent with those data, and they highlight the importance of cardiac cAMP catabolism in regulating cardiac contractility. PDE4 is associated with many intracellular complexes involved in β-adrenergic signaling, calcium signaling with the ryanodine receptors and L-type calcium channels (LTCCs), contraction associated to myofilaments [42] and lastly I_Ks_ potassium channel [43]. PDE2 is also associated with β-adrenergic signaling complexes and LTCCs. Although the activity of PDE2 toward the hydrolysis of cAMP is relatively low, its presence in plasma membrane contributes to regulate the cardiac LTCC activity when the level of cGMP is increased [44], since PDE2 inhibits the activation of LTCCs by reducing cAMP concentration after being activated by cGMP. By this mechanism, PDE2 establishes a link in the heart between cGMP generated by guanylyl cyclases and β-adrenergic cAMP signal [45].

Only rats undergoing a two week-treatment with angiotensin II at 0.4 mg.kg^−1^.d^−1^ developed hypertension associated with cardiac hypertrophy. However, this cardiac hypertrophy was weak (+24%) indicating that cardiac hypertrophy is in its early stage.

We observed a specific increase in PDE4 activity, which results of opposite changes. Indeed, while all detected isoforms of the PDE4D subfamily were down-regulated, we observed a specific increase in a short 69 kDa-PDE4A isoform by angiotensin II treatment, without increase in PDE4A mRNA. This apparent discrepancy is due to the fact that the set of primers used were designed to detect all PDE4A mRNAs while three variants were separated by Western blot. PDE4A seems to be the major subfamily responsible for cAMP hydrolysis in rat left cardiac ventricle. It has been shown in isolated rat ventricular myocytes that PDE4A contribution was one third of total PDE4 activity, while PDE4D accounted only for 15% [46]. This explains in part why, despite a decreased PDE4D expression, global cAMP-PDE4 activity is increased. Little is known about the distribution and role of PDE4A in heart. It was shown that PDE4A1 could be associated to the Golgi membrane [47]. On the other hand, PDE4D has already been linked to cardiac diseases and its role in heart was intensively investigated. Several epidemiologic studies found an association between PDE4D gene and ischemic stroke [48], [49]. PDE4D deficiency in the ryanodine-receptor complex (RyR_2_) in mice was also involved in heart failure. Accumulation of cAMP contributes to PKA-hyperphosphorylated RyR2 channels leaking calcium that promotes cardiac dysfunction and arrhythmias [14]. PDE4D3, which is the isoform supposed to be in the ryanodine-receptor complex, is also associated to sarcoplasmic [50] and perinuclear [51] membranes. Furthermore, PDE4D was shown to be associated to isolated cardiac nuclei [52]. Moreover, PDE4D5 is implicated in β_2_-adrenergic receptors signaling through β-arrestin interactions [53]. Our data are consistent with those results and show moreover that this PDE4D decrease occurs at a very early stage in the development of cardiac hypertrophy.

The highest cGMP-PDE effect of angiotensin II treatment at 0.4 mg.kg^−1^.d^−1^ was on PDE1 activity in its basal state, with a 130% increase. The expressions of 110 kDa-PDE1A, and 67 kDa- and 73 kDa-PDE1C proteins were similarly increased by angiotensin II treatment. But in the same time, PDE1 activation by Ca^2+^/CaM complexes was less important than in control rats. This could be the result of a PKA-dependent phosphorylation. Indeed, PKA-dependent phosphorylation of PDE1A and PDE1C has been shown to reduce their ability to get activated by Ca^2+^/CaM complexes [54]. However, considering cAMP hydrolysis activity of PDE1 which concerned PDE1C (**Fig. 5C**), no difference in PDE1 ratio was observed, suggesting that during cardiac hypertrophy, PDE1A was specifically PKA-phosphorylated. An increase in PDE1 activity and PDE1A expression has been reported to be associated with cardiac hypertrophy induced by abdominal aortic banding in Sprague-Dawley rats [12], and more recently by neurohumoral stimuli such as angiotensin II and isoproterenol [55]. Interestingly, it was shown in cultured rat aortic vascular smooth muscle cells that angiotensin II treatment upregulates PDE1A [56], suggesting that such mechanism could participate to PDE1A upregulation described herein. PDE1A hydrolyzes mainly cGMP and PDE1C hydrolyzes cGMP and cAMP equally. PDE1C is expressed in rat heart [57] and PDE1C1 is expressed at high levels in human cardiac myocytes [58]. PDE1C has been associated with arterial smooth muscle cell proliferation in several studies [59], [60]. Interestingly, an increase in PDE1 activity and PDE1C protein was proposed to explain in part the cardioprotective effect of prostacyclin in rat heart [57]. Thus, to our knowledge, it is the first time that, additively to PDE1A, PDE1C is shown to be associated with cardiac hypertrophy in the heart.

Angiotensin II treatment induced a 113% increase in PDE5 activity, corresponding to strong increases in both 83 kDa- and 97 kDa-PDE5A isoforms and a 121% increase in PDE5A-mRNA. This is in agreement with the reported increase of PDE5A expression induced by angiotensin II in vascular smooth muscle cells representing a new mechanism by which angiotensin II antagonizes cGMP signaling [61]. In that way, PDE5 activity and expression have been reported to be elevated in right ventricular hypertrophy in rats as well as in humans [21], and cardiac hypertrophy induced by aortic banding in C57Bl/6 mice [22]. On one hand, cardiac over-expression of PDE5A in mice predisposed to left ventricle remodeling [15], and interestingly PDE5A inactivation by gene silencing blunted phenylephrine-induced hypertrophy [62]. On the other hand, PDE5 inhibition improved cardiac contractility [21], [63], and prevented and reversed cardiac hypertrophy [22]. This effect is mediated by one member of the Regulator of G-protein Signaling family RGS2 [64]. All these results brought increasing interest in using PDE5 inhibitors in the treatment of heart failure [65]. Recently, it was shown in the heart that: i) PDE5A is coupled to NOS3; ii) this coupling targets PDE5A to a specific intracellular location (Z-bands) enabling PDE5A to regulate cGMP generated through NOS3 activation in response to β-adrenergic stimulation; and iii) chronic inhibition of NOS3 abolishes this localization [36]. Herein, we show that NOS3 expression is not affected in early cardiac hypertrophy induced by angiotensin II and that PDE5A keeps a Z-band localization pattern. Taken together, our data additively show that PDE5A alterations begin at a very early stage in cardiac hypertrophy leading to heart failure, and that the reported increase in PDE5 activity occurs before the loss of its intracellular localization to Z-bands and alteration of NOS3 expression.

In agreement with PKG activity suppression reported in hypertrophied human right ventricle [21], a reduction in PDE5A Ser92 phosphorylation was observed herein indicating a decrease in PKG activity, which might result from increased cGMP-PDE1, -PDE2, and -PDE5 activities. Indeed, it was proposed that PDE5 phosphorylation could be used as an *in vivo* indicator for PKG activation [66]. Furthermore, PDE5A up-regulation might decrease the cGMP binding to PDE5A GAF domain and consequently its phosphorylation [67]. These data support a decrease in the availability of cGMP in the vicinity of PDE5, most likely to Z-bands.

PDE regulation is complex and one transduction signal may affect several PDEs. For example, cAMP differentially regulates PDEs through PKA by activating PDE4 and decreasing PDE1 activation [68]. Regarding cGMP, we could think that PDE1 and PDE5 may have a common pattern of regulation in cardiomyocytes. Indeed, they share some common intracellular localization like the Z-bands [36], [58], [69] and participate to contractile events [70], [71]. They are implicated also in cell growth and survival with nuclear localizations for PDE1A [71] and PDE1C [69] and centrosomal localization for PDE5A [69]. Their activities were simultaneously increased in vascular smooth muscle cell proliferation [60], [70] and herein in cardiac hypertrophy. Their increases might participate together to nitrate tolerance, since combination of nitrates with PDE5 inhibitors [72] or PDE1 inhibitors [73] improved pulmonary vasodilator response in pulmonary arterial hypertension. It was also suggested that sildenafil improved vasodilatation in pulmonary hypertension through PDE5 and PDE1 inhibitions [74]. Very few data are available in cardiac myocytes due to lack of simultaneous studies on PDE1 and PDE5.

Angiotensin II treatment at 0.4 mg.kg^−1^.d^−1^ induced a 76% increase in cGMP-PDE2 activity in cardiac ventricles without any change in expression. This suggests that increased PDE2 activity was the result of a post-translational regulation. Such increased PDE2 activity, without increased expression, has previously been reported in another rat cardiac hypertrophy model induced by abdominal aortic banding [12]. It was shown that PDE2 could be activated by protein kinase C (PKC)-dependent phosphorylation [75]. Since PKC is part of the AT1 receptor signaling pathway and is likely to be activated after angiotensin II stimulation, the increase in PDE2 activity subsequent to angiotensin treatment might be PKC-mediated [5]. In rat ventricular cardiomyocytes, stimulation of soluble or particulate GC to the production of cGMP in different functional compartments, which access is restricted to different cGMP-PDEs. For example, cGMP produced by particulate GC would be exclusively under the control of PDE2, whereas that produced by soluble GC would be under the control of PDE2 and PDE5 [76]. We observed an up-regulation of the brain natriuretic peptide which stimulates its receptor, through an autocrine/paracrine action, increasing the “particulate” cGMP pool [7]. It was reported that natriuretic peptides could inhibit angiotensin II-induced proliferation of rat cardiac fibroblasts through cGMP-dependent mechanism [77]. The increased PDE2 activity observed might then oppose the BNP-induced cGMP increase, which could blunt its cardioprotective effect. Moreover, it was shown that natriuretic peptide desensitization in heart failure relates, in part, to increased PDE5 activity, supporting a therapeutic role for PDE5 inhibition [78]. Furthermore, stimulation of isolated cardiomyocytes by phenylephrine induced hypertrophy, and PDE5A over-expression amplified natriuretic peptide gene expression from phenylephrine stimulation [62]. It is the first time that such PDE2 activation is reported in angiotensin II induced hypertrophy, strengthening previous data in aortic banding-induced cardiac hypertrophy in Sprague-Dawley rats [12].

As a direct consequence of increased PDE4 activity which, degrades cAMP, the increase of 5′AMP could lead to AMPK activation through an increase in AMP/ATP ratio. This hypothesis is supported by a recent work showing that IL-6 activates AMPK in skeletal muscle by transiently increasing the concentration of cAMP and, secondarily, the AMP/ATP ratio [79]. AMPK is a heterotrimeric protein consisting of a catalytic α subunit and β and γ regulatory subunits and is a serine/threonine protein kinase [80]. It is a key metabolic regulator that is activated under stress conditions. Its exact role in cardiac diseases is until now not well defined, but in recent studies an overall cardioprotective role of AMPK was suggested [37], [81]. Indeed, AMPK activation with AICAR (5-amino-4-imidazolecarboxamide riboside, an AMPK activator) inhibited growth and proliferation in rat neonatal cardiac fibroblasts [82]. AMPKα2 deficiency was shown to enhance myocardial ischemia/reperfusion injury in a mouse model with cardiomyocyte-specific overexpression of a mutant AMPKα2 catalytic subunit [83], and to exacerbate pressure overload induced left ventricular hypertrophy and dysfunction in AMPKα2 knock-out mice [84]. Direct and indirect activation of AMPK was also shown to have cardioprotective properties. Metformin was able to induce AMPK phosphorylation and to improve left ventricular function and survival in heart failure [85]. Resveratrol was also shown to inhibit cardiac hypertrophy in part through AMPK activation [86]. In rat neonatal cardiomyocytes, angiotensin II-induced hypertrophy was accompanied by a decreased activation of AMPK, and stimulation with the AMPK-activator AICAR inhibited this hypertrophy [87]. Long-term activation of AMPK with AICAR was also shown to attenuate cardiac hypertrophy induced by pressure overload [88]. Moreover, a recent study showed that the anesthetic sevoflurane induced AMPK activation protects the heart against ischemia and reperfusion injury, and relies on upstream production of ROS [89]. In our cardiac hypertrophy model, AMPK phosphorylation was increased by 64%. This activation might be the result of increased generation of 5′AMP by PDE4 activity, and therefore might be a physiological mechanism to oppose left ventricular hypertrophy induced by angiotensin II.

The implication of NOX and ROS in cardiac remodeling has been well documented (for review see [90], [91]). For example, it was shown in rat neonatal cardiomyocytes that NOX2 [92] is implicated in the cardiac hypertrophic effect of angiotensin II. Surprisingly, we observed a 66% decrease in the NOX2 mRNA expression. However, it was recently shown that natriuretic peptides such as ANP have antihypertrophic effect on cardiomyocytes, in part by counteracting increased ROS generation and NOX2 expression induced by angiotensin II [93]. Moreover, a recent study showed that AMPKα2 subunit functions as a physiological suppressor of NAD(P)H oxidase (NOX) and ROS production in endothelial cells [94]. It was also suggested that AMPK may enhance NO bioavailability indirectly through antioxidative mechanisms by limiting superoxide-triggered NO consumption and consecutive peroxynitrite (ONOO^-^) production, e.g., by inhibiting NOX [95], [96]. We could then make the assumption that the increased BNP and AMPK in our model may have a negative effect on NOX2 expression, at least in the early stages of cardiac hypertrophy development.

**Figure 9:**
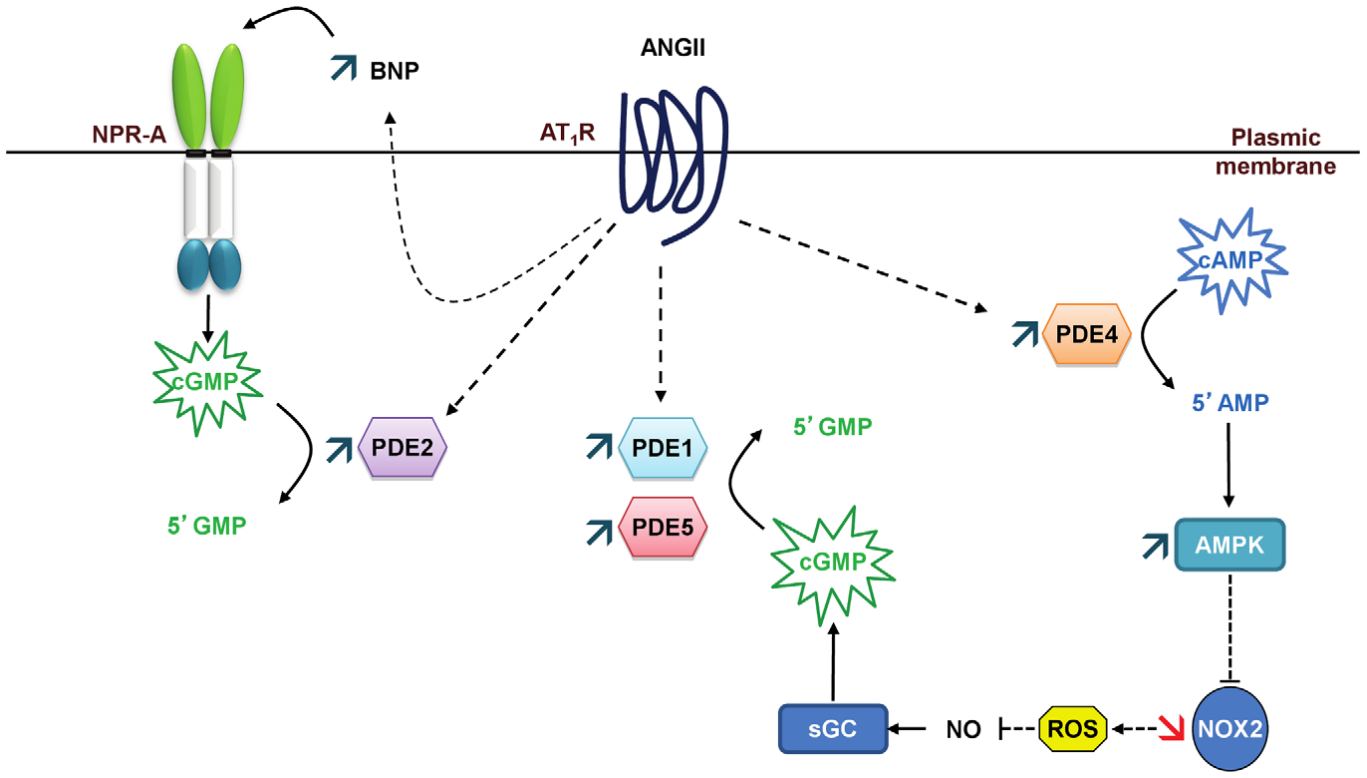
Proposed cardioprotective mechanisms that could lead to increase cGMP. The expression of natriuretic peptides could increase “particulate” cGMP pool, and the activation of AMPK, subsequent to increased PDE4 activity, could increase “soluble” cGMP pool through enhanced NO bioavailability. These increases in cGMP could also have effects on PDE activities: “particulate” cGMP may contribute to further increase PDE2 activation and “soluble” cGMP may further activate PDE5, enhancing even more cGMP hydrolysis.

Cardiac hypertrophy and its progression to heart failure represent a major risk of sudden death. We show in this work that the early stages of cardiac hypertrophy induced by angiotensin II are associated with deep alterations in cAMP- and cGMP-PDE activities. While a specific rise in cAMP-PDE4 activity is observed, cGMP-PDEs activities (PDE1, PDE2 and PDE5) are broadly increased favoring a greater cGMP hydrolysis. This result is of particular interest knowing that previous works on other models focused either on cAMP- or cGMP-PDEs. PDE4D is strongly down-regulated, while PDE1A and PDE5A are highly up-regulated. Each of these alterations has already been reported in advanced cardiac hypertrophy and heart failure. Herein we show that these changes occur and vary in an opposing manner from the very beginning of the development of cardiac hypertrophy, confirming their potential role as targets in cardiac hypertrophy prevention. Moreover, we highlight for the first time that PDE1C is present in left cardiac ventricle and increased in early stages of cardiac hypertrophy. The role of PDE1 family in the heart has only very recently raised interest and is still not well known. Furthermore, we report that PDE2 accounts for the main contribution in cGMP-PDE activity in rat left cardiac ventricle and that PDE2 activity increases in the early stages of cardiac hypertrophy through a post-translational regulation. Finally, associated with the global increased cGMP hydrolysis in hypertrophic left cardiac ventricle, we observe the establishment at the PDE levels of two cardioprotective mechanisms and we suggest that these mechanisms could lead to increase cGMP: i) expression of natriuretic peptides could increase “particulate” cGMP pool; ii) activation of AMPK, subsequent to increase in PDE4 activity, could increase “soluble” cGMP pool through enhanced NO bioavailability (**Fig. 9**). More studies are needed to support these assumptions. Nevertheless, our results suggest a potential link between PDE4 and AMPK/NOX2 and that cGMP-PDEs, especially PDE1 and PDE2, may be interesting therapeutic targets in preventing cardiac hypertrophy.
